# The 5‐HT_2C_ receptor as a therapeutic target for alcohol and methamphetamine use disorders: A pilot study in treatment‐seeking individuals

**DOI:** 10.1002/prp2.767

**Published:** 2021-04-30

**Authors:** Erin J. Campbell, Yvonne Bonomo, Adam Pastor, Lisa Collins, Amanda Norman, Peter Galettis, Janice Johnstone, Andrew J. Lawrence

**Affiliations:** ^1^ Florey Institute of Neuroscience and Mental Health Melbourne Brain Centre The University of Melbourne Parkville VIC Australia; ^2^ Department of Addiction Medicine St Vincent's Hospital Melbourne The University of Melbourne Parkville VIC Australia; ^3^ School of Medicine and Public Health The University of Newcastle Callaghan NSW Australia

**Keywords:** 5‐HT_2C_ agonist, alcohol, craving, lorcaserin, methamphetamine, serotonin

## Abstract

Alcohol use disorder (AUD) and methamphetamine use disorder (MUD) are prevalent and have high adverse impacts on both the individual and society. Current treatment strategies for these disorders are ineffective at a population level. Lorcaserin, a 5‐HT_2C_ receptor agonist, has shown potential at reducing the symptoms of substance use disorder. This pilot study (initiated prior to market withdrawal) examined feasibility and safety of lorcaserin treatment in people undergoing residential detoxification and treatment for AUD and MUD. This was an open label pilot study of lorcaserin where participants (*n* = 10 AUD; *n* = 8 MUD) received 10‐mg lorcaserin daily for 4 days then twice daily for 1 month. Primary outcome measures included recruitment and retention rate, incidence of treatment‐emergent events, incidence of methamphetamine or alcohol withdrawal‐related events, heart rate, and blood pressure. Secondary measures included pharmacokinetic data and self‐reported alcohol or methamphetamine use, craving, and psychological distress. AUD participants were recruited faster and had a greater retention rate compared with MUD participants. Lorcaserin did not alter vital signs, was well tolerated, and had a similar pharmacokinetic profile to individuals with obesity. Lorcaserin reduced self‐reported alcohol and amphetamine‐type substance use and craving in AUD and MUD participants, respectively. Self‐reported psychological health also improved over the treatment period for all participants. Despite the pilot nature of this study, our data support the notion of 5‐HT_2C_ receptors as a therapeutic target for drug and alcohol abuse.

**Abbreviations**
AUD: Alcohol use disorder
BMI: body mass index
FDA: Food and Drug Administration
MUD: methamphetamine use disorder

## 1 INTRODUCTION

Alcohol use disorder (AUD) poses a major social and economic burden to society, accounting for ~5% of deaths worldwide in 2016 and costing upward of $36 billion/year in Australia.^1^ With regard to illicit drug use, methamphetamine is the second most regularly used drug following cannabis in Australia, and in the United States, there was a threefold increase in the number of methamphetamine‐associated deaths between 2010 and 2015.^2^, ^3^ As a result, the recreational use and abuse of drugs continues to be a major global public health issue. Importantly, poor retention and frequent relapse remain serious obstacles for the treatment of substance use disorders with continuous and intense cravings persisting both during and following treatment.^4^, ^5^ There are three Food and Drug Administration (FDA)‐approved pharmacotherapies for AUD, disulfiram, naltrexone, and acamprosate. Current pharmacotherapeutic treatments for AUD remain ineffective at a population level due to a combination of limited effectiveness and under‐prescribing.^6^, ^7^ There are no FDA‐approved pharmacotherapies specifically for methamphetamine use disorder (MUD). In a recent review, most medications evaluated were found to not have a statistically significant benefit.^8^ Clearly, there is need for improved pharmacotherapeutic treatments aimed at reducing drug‐related craving for both AUD and MUD.

The neural circuitry surrounding craving and relapse to drug use involves several brain structures including the ventral tegmental area, striatal complex, amygdala, hippocampus, and the prefrontal cortex.^9^, ^10^ Serotonin‐containing cells (5‐hydroxytryptamine [5‐HT]) are located predominantly in the raphe nuclei of the mid/hind‐brain, including the dorsal and median raphe.^11^ From here, 5‐HT fibers widely innervate the central nervous system including multiple nodes of reward‐related circuitry.^12^ Indeed, the serotonergic system has been implicated in substance use disorder and relapse for several decades and may represent an avenue for future pharmacological interventions.^13^, ^14^, ^15^, ^16^, ^17^, ^18^ There are 14 known subtypes of 5‐HT receptors, and serotonin signaling is well known to modulate dopamine activity.^19^ Specifically, the 5‐HT_2C_ receptor is expressed in the hippocampus, striatum, and amygdala in both rat and human.^20^, ^21^, ^22^

5‐HT_2C_ receptor signaling has been implicated in the development and maintenance of AUD and MUD. For example, the 5‐HT_2C_ receptor agonist, Ro60‐0175, decreased alcohol consumption in rats whereas the 5‐HT_2C_ receptor antagonist, SB‐242 084, increased alcohol consumption.^23^ However, Ro60‐0175 reduced both alcohol (gel solution) and vehicle (plain gel containing polycose) operant self‐administration in rats suggesting possible nonspecific effects on caloric intake.^24^ 5‐HT_2C_ receptor signaling is also involved in methamphetamine use; for example, Ro60‐0175 reversed methamphetamine self‐administration‐induced decreases in nucleus accumbens shell excitability.^25^ Additionally, methamphetamine‐induced behavioral sensitization is associated with a functional upregulation of 5‐HT_2C_ receptors in the ventral pallidum.^26^ Together, these preclinical studies highlight a role for the 5‐HT_2C_ receptor in both alcohol and methamphetamine use.

The development of therapeutic drugs that selectively target individual 5‐HT_2_ receptor subtypes is difficult. Indeed, most 5‐HT_2C_ receptor agonists also bind to 5‐HT_2A_ and/or 5‐HT_2B_ receptors.^27^ Lorcaserin is a selective serotonin 2C (5‐HT_2C_) receptor agonist with a 3‐benzazepine scaffold,^28^ developed as an anti‐obesity medication. Notably, lorcaserin reduces the consumption of alcohol in rats and methamphetamine use in rhesus monkeys,^29^, ^30^ implicating the 5‐HT_2C_ receptor as a potential treatment target for alcohol and substance use disorders.^15^ Until recently, lorcaserin (Belviq^®^) was FDA‐approved as an anti‐obesity medication ^31^ but was withdrawn from the market after a safety trial indicated an increased occurrence of cancer, where 7.7% of participants receiving drug developed cancer relative to 7.1% in the placebo arm.^31^ Here, we carried out a pilot study, prior to market withdrawal, to evaluate the ability of lorcaserin to suppress alcohol and methamphetamine craving and consumption in treatment‐seeking AUD or MUD participants.

## 2 MATERIALS AND METHODS

### 2.1 Study design and participants

This was an open label pilot study. The protocol and amendments were approved by the Human Research Ethics Committee of St Vincent's Hospital Melbourne (HREC 031/17). Eligible participants were males and females over the age of 18 years, diagnosed with alcohol or methamphetamine substance use disorder (DSM5). Exclusion criteria included pregnant (urine βHCG positive) or breastfeeding; highly dependent on medical care for co‐existing conditions; other medical treatments for substance dependence including anti‐craving (e.g., acamprosate and naltrexone), aversive (e.g., disulfiram), or substitution (e.g., atomoxetine, dexamphetamine, and methylphenidate) treatments; known allergy to lorcaserin; already receiving lorcaserin; severe liver impairment (Child Pugh C); severe renal impairment (creatinine clearance <30 ml/min); hypertension; unstable diabetes; history of serotonin syndrome; low body mass index (BMI <20); and unstable mental state (including active psychosis or schizophrenia). Written informed consent was obtained from all participants.

The study protocol specified the recruitment of 10 AUD participants and 10 MUD participants. Recruitment was ceased when the initial FDA alert was issued in January 2020, resulting in the final recruitment of 10 alcohol‐ and 8 methamphetamine‐dependent participants. Note that the HREC was advised immediately by the Chief Investigators of the original FDA alert and the subsequent product withdrawal. All participants were advised in writing of the product withdrawal and the health risks associated with their participation in the study.

### 2.2 Procedures

Participants were treated with the lowest effective dose of lorcaserin (immediate release) used in the treatment of obesity. Participants received lorcaserin 10 mg once daily for 4 days then twice daily for 1 month. All participants received symptomatic treatment using the St Vincent's Hospital standard protocol. Withdrawal was actively managed as either an outpatient or, if required, in a residential setting. Alcohol withdrawal was managed in accordance with Clinical Institute Withdrawal Assessment of Alcohol (CIWA‐Ar).^32^ Prescribed medications consisted of 5–20 mg of diazepam 2 hourly with a maximum of 80 mg per 24‐h period, anti‐emetics for nausea, and paracetamol and nonsteroidal anti‐inflammatories for aches. All medications were used only as required and ceased by Day 7 following participants’ last use of alcohol or methamphetamine and the commencement of lorcaserin.

### 2.3 Pharmacokinetic sampling

On Day 7 of treatment, 4‐ml EDTA blood samples were taken for assessment of plasma concentration levels of the IP (lorcaserin) at predose (time 0), 2, 4, and 8 h after treatment. Bloods were refrigerated at ≤4°C and centrifuged at 2000*g* for 10 min within 12 h after blood sampling. Immediately after centrifugation, plasma were stored in two labeled polypropylene tubes and stored at ≤−20°C for plasma concentration analysis. All plasma concentration analyses were performed after all participants had completed the final visit.

### 2.4 Measures

Measures were assessed over five treatment time points (baseline, Day 7, Day 14, Day 21, and Day 28). A baseline researcher‐administered questionnaire assessed demographic and clinical characteristics and determined eligibility. Liver function and blood glucose were assessed every second week (baseline, Day 14, and Day 28).

The Obsessive Compulsive Drinking Scale was used to assess alcohol craving.^33^ A total craving score was calculated by summing the 14 items of this questionnaire, which were ranked on a Likert scale ranging from 1 to 7. Summing Items 1–6 calculated the obsessive subscale, and summing Items 7–14 calculated the Compulsive Subscale. A brief, 10‐item Methamphetamine Craving Questionnaire, based on the Cocaine Craving Questionnaire, was used to assess methamphetamine craving over time. Scores across each item were averaged.^34^, ^35^

The Kessler Psychological Distress Scale (K10) yielded a global measure of distress, which was the sum of all 10 items.

The Australian Treatment Outcomes Profile (ATOP), a validated Australian version of the UK Treatment Outcome Profile, was used to assess self‐reported drug use and health and well‐being.^36^ Higher scores on the substance use questions, measured using the timeline follow‐back method, reflected more days of use whereas higher scores on health and well‐being questions indicated greater self‐rated health outcomes.

### 2.5 Outcomes

Primary endpoints were prespecified. Feasibility endpoints were recruitment rate and retention in treatment at Days 7, 14, 21, and 28. Safety endpoints were incidence of treatment‐emergent adverse events (AEs); incidence of methamphetamine or alcohol withdrawal‐related treatment‐emergent events; heart rate; and blood pressure.

Secondary endpoints were methamphetamine use (self‐report, saliva screen, and urine drug screen) on Study Days 0, 7, 14, 21, and 28; alcohol use (self‐report, breath alcohol, blood testing, and urine drug screen) on Study Days 0, 7, 14, 21, and 28; craving measures—Obsessive Compulsive Scale for Drinking (AUD group) and Cocaine Craving Questionnaire (MUD group) on Study Days 0, 7, 14, 21, and 28; and K10 psychological distress and ATOP measures on Study Days 0, 7, 14, 21, and 28.

A final outcome was to assess the pharmacokinetics of lorcaserin in a clinical population with normal BMI: AUC 0–8 h, *C*_max_, *t*_½_, *t*_max_.

### 2.6 Pharmacokinetic analysis

Pharmacokinetic data were analyzed at the Clinical Pharmacology Laboratory, School of Medicine and Public Health, University of Newcastle, Australia. Plasma samples were analyzed using a validated liquid chromatography with tandem mass spectrometry (LCMSMS) method for lorcaserin. Plasma samples (50 µl) were prepared by adding twice the volume of acetonitrile, samples were vortexed and then centrifuged, and the supernatant was transferred to a vial and injected onto the LCMSMS. The LCMSMS system consisted of a Shimadzu 8060 LCMS using a Kinetex C18 column and a gradient of 0.1% formic acid and acetonitrile. Lorcaserin was linear over the range of 10–500 ng/ml. Using the concentration–time profiles determined by the analysis of the plasma samples, the pharmacokinetic parameters, maximum plasma concentration (*C*_max_), time of *C*_max_ (*T*_max_), area under the plasma concentration time curve (AUC 0–12), and plasma half‐life (*t*_½_), were determined using PKSolver.^37^ The data were fitted using the NCA Extravascular module, with AUC_0 −_ *_t_* calculated using the log‐linear trapezoidal method. The 12‐h time point for each individual was predicted with a one compartment model using PKSolver.

### 2.7 Statistical analysis

Unless otherwise stated, data were analyzed separately for AUD and MUD participants.

Demographic data were summarized as numbers and proportions for categorical data and mean (±standard error of mean [SEM]) for continuous data using Microsoft Office Excel 2016. Clinical characteristics and vital signs were analyzed using a repeated measures ANOVA. Due to the high dropout rate of participants with MUD, either three (baseline to Day 14) or five time points (baseline to Day 28) were assessed. The oral fluid test and urinary drug screen were analyzed using the Friedman nonparametric test. For participants with AUD, baseline and Day 21 oral fluid substance results were not detected for one participant and baseline blood glucose levels were missing for another participant. For participants with MUD, Day 7 blood pressure data were missing for one participant, baseline weight data were missing for two participants, and Day 7 weight data were missing for one participant. To allow for the inclusion of these participants in statistical analyses, a mean imputation of missing values was conducted in SPSS. For MUD participants, one participant had all liver function and blood glucose data missing and three participants had their urine drug screen data missing and were thus excluded from analyses. The Obsessive Compulsive Drinking Scale, Methamphetamine Craving Questionnaire, and K10 Scale were analyzed using a repeated measures ANOVA as the data were normally distributed and ANOVA assumptions were not violated. A repeated measures ANOVA was used to analyze the ATOP data. For participants with AUD, the ATOP data for the number of days participants drank alcohol and the typical quantity of alcohol consumed were not normally distributed and thus assessed using the Friedman nonparametric test. Two participants had data missing from the health and well‐being questions and were excluded from this analysis. Only two to three participants with MUD completed the ATOP self‐report questionnaire for the duration of the study (baseline to Day 28); thus, data were also analyzed from baseline to Day 14, with five participants completing the questionnaire at these time points. All analyses were performed using SPSS v27 (*α* = .05). Data are presented as mean ± SEM.

### 2.8 Nomenclature of targets and ligands

Key protein targets and ligands in this article are hyperlinked to corresponding entries in http://www.guidetopharmacology.org, the common portal for data from the IUPHAR/BPS Guide to PHARMACOLOGY,^38^ and are permanently archived in the Concise Guide to PHARMACOLOGY 2019/20.^39^

## 3 RESULTS

### 3.1 Demographic information

The participants recruited with AUD were predominantly male (8/10). The mean age was 48.1 years (SEM = 2.1). Mean typical quantity of alcohol consumed on day used was 21.8 standard drinks (SEM = 4.4). The participants recruited with MUD were predominantly male (6/8) with a mean age (*M* = 36.6 years, SEM = 2.9) of more than 10 years younger than the AUD group. The mean typical quantity of methamphetamine on a day used was 0.66 g (SEM = 0.22).

Table 1 describes further characteristics at baseline for both participant groups. Age of use characteristics were younger for AUD participants compared with MUD participants. AUD participants had experienced more withdrawal symptoms compared with MUD participants. The majority of AUD participants (90%) had at least one previous treatment for dependence compared with 50% for MUD participants. The types of treatments for AUD were both pharmacotherapies and nonpharmacotherapies, but only nonpharmacotherapies for MUD participants.

**TABLE 1:** Participant characteristics at baseline

Characteristic	Number (%)	
	Alcohol use disorder (*n* = 10)	Methamphetamine use disorder (*n* = 8)
Country of birth		
Australia	8 (80.0)	6 (75.0)
Other	2 (20.0) (Yugoslavia, United Kingdom)	2 (25.0) (Scotland, South Africa)
Indigenous Australian	1 (10.0)	0
Living status		
Alone	4 (40.0)	2 (25.0)
Partner (with at least 1 child)	3 (30.0)	2 (25.0)
Partner (no children)	2 (20.0)	1 (12.5)
Relationship status		
Single	4 (40.0)	5 (62.5)
Living with spouse (including married, de facto, life partner)	4 (40.0)	3 (37.5)
Separated but not divorced	2 (20.0)	0
Accommodation		
Private accommodation	10 (100.0)	8 (100.0)
Employment		
Employed	4 (40.0)	4 (80.0)
Disability support pension	2 (20.0)	1 (12.5)
Currently unemployed	1 (10.0)	1 (10.0)
Highest level of education		
Secondary (years 7–10)	2 (10.0)	3 (37.5)
Secondary (years 11–12)	1 (10.0)	3 (37.5)
Tertiary	6 (60.0)	1 (12.5)
Post graduate	1 (10.0)	1 (12.5)
Medical history		
Current conditions		
Depression	4 (40.0%)	3 (37.5%)
Anxiety	4 (40.0%)	3 (37.5%)
High cholesterol	2 (20.0%)	0
Alcohol‐related conditions		
None	7 (70.0%)	6 (75.0%)
Seizures	2 (20.0%)	0

Alcohol or methamphetamine use history	Alcohol use	Methamphetamine use
	Estimated amount (standard drinks)	Estimated amount (g)
Mean (SE)	21.8 (4.4)	0.66 (0.22)
	Age at first drink (years)	Age at first use (years)
Mean (SE)	16.3 (0.4)	24.0 (3.1)
	Age at daily drinking (years)	Age at problematic use (years)
Mean (SE)	31.6 (2.8)	25.7 (3.4)
	Duration since daily drinking (years)	Duration since problematic use (years)
Mean (SE)	15.8 (2.5)	10.4 (2.5)
Previous detoxifications		
At least 1 detoxification	9 (90.0%)	4 (50.0%)
Previous withdrawal symptoms		
Sweating	9 (15.0%)	1 (1.7%)
Anxiety	8 (13.3%)	3 (5.0%)
Tremor	7 (11.7%)	1 (1.7%)
Nausea	5 (8.3%)	0
Agitation	5 (8.3%)	3 (5.0%)
Seizures	2 (3.3%)	0
Vomiting	3 (5.0%)	0
Diarrhea	2 (3.3%)	0
Delirium tremens	1 (1.7%)	0
Previous treatment for dependence		
At least 1 treatment	9 (90.0%)	4 (50.0%)
Previous types of treatment (often multiple)		
Counseling	9 (100.0%)	4 (100.0%)
Acamprosate maintenance	9 (100.0%)	0
Naltrexone maintenance	8 (88.9%)	0
Alcoholics anonymous (AA)	7 (77.8%)	0
Residential rehabilitation activities	4 (44.4%)	1 (25.0%)
Disulfiram maintenance	2 (22.2%)	0
Other	3 (33.3%) (recovery and support program, 2× baclofen)	1 (25.0%) (The Rewired Program—self‐administered)

### 3.2 Primary endpoints

#### 3.2.1 Feasibility

The first participant was recruited into the clinical trial on the 24th of July 2018, and the last participant was recruited on the 2nd of December 2019, equating to a total of just over 16 months (496 days) to recruit the 18 participants. For the alcohol group (*n* = 10), the median time between recruiting each participant was just over 1 month (median = 37 days, interquartile range = 16–59 days), that is, a rate of 0.82 AUD participants per month. For the MUD group (*n* = 8), it took longer between participants to be recruited (median = 73 days, interquartile range = 44–98 days), that is, a rate of 0.42 participants per month. For the AUD participants, the retention in treatment rate at Day 7 was 100%, and 80% for each weekly visit thereafter. For the MUD participants, the retention rate at Days 7, 14, 21, and 28 was 87.5%, 62.5%, 37.5%, and 50%, respectively. The main reason for participants discontinuing the trial was due to failing to attend appointments. Note that one patient missed Day 21 appointment but represented for Day 28, and this accounts for the dip in the retention rates over time at Day 21. Each was deemed “lost to follow‐up” after multiple unsuccessful attempts by a clinician to contact the participant.

#### 3.2.2 Safety

##### Treatment‐emergent AEs

Eight AUD participants (80%) reported at least one AE, and seven MUD participants (87.5%). Of these, seven of the AUD participants (87.5%), and five MUD participants (71.4%), reported at least one AE that was considered by a study doctor to be related to lorcaserin. These included ratings of definitely related, probably related, or possibly related (Table 2). Of all AEs (*n* = 28), 19 were considered to be treatment emergent (67.9%).

**TABLE 2:** Number of treatment‐emergent adverse events (*n* = 19) (definitely related, probably related, or possibly related to lorcaserin) grouped by System Organ Class and preferred term, for both groups of participants

Event (System Organ Class/preferred term)	Alcohol use disorder (*n* = 10)	Methamphetamine use disorder (*n* = 8)
Number of participants with ≥1 medication‐related adverse event	7	5
Gastrointestinal disorders		
Diarrhea	0	1
Dry mouth	2	1
Lip dry	1	0
Nausea	0	1
General disorders and administration site conditions		
Lethargy	3	0
Metabolism and nutrition disorders		
Decreased appetite	3	1
Nervous system disorders		
Dizziness	2	0
Headache	2	2

The most frequently occurring treatment‐emergent AEs were “decreased appetite” (21.1%, 4 of 19) and headache (21.1%, 4 of 19). These occurred throughout the 4 weeks of study medication administration. The second most frequently occurring treatment‐emergent AEs were “lethargy” (15.8%, 3 of 19) and “dry mouth” (15.8%, 3 of 19), again occurring through the duration of the trial. None of the treatment‐emergent AEs was classified as “severe.” One was classified as “moderate” severity. This event of headache reported at Day 21 lasted 2 days and resolved spontaneously. The remaining 18 treatment‐emergent AEs were classified as “mild” severity. None of the treatment‐emergent AEs was a serious AE.

##### Withdrawal‐related treatment‐emergent AEs (*n* = 1)

One event was “probably related” to lorcaserin. An AUD participant experienced ongoing fatigue. They dropped out at Day 9, and no further follow‐up by the study staff was achieved.

##### Vital signs

For participants with AUD, temperature, pulse, systolic and diastolic blood pressure, and weight did not significantly change over time (Table S1). Respiratory rate (breaths/min) significantly increased over the treatment period (Table S1). For participants with MUD, temperature, pulse, respiratory rate, systolic and diastolic blood pressure, and weight did not significantly change over time (Table  S2).

### 3.3 Secondary endpoints

#### 3.3.1 Substance use

##### Alcohol use

###### Self‐report

For the AUD group, the number of days drinking alcohol in the past week did not change between baseline and Day 28 (Table 3); however, the typical quantity of alcohol consumed on any given day did decrease over time (Table 3).

**TABLE 3:** ATOP self‐report data from baseline to Day 28 for participants with alcohol use disorder and methamphetamine use disorder

ATOP self‐report	*F* statistic	*χ* ^2^	*p* value	*n*
Alcohol use disorder participants				
Number of days drank alcohol in the last 7 days		5.756	.218	8
Typical quantity (standard drinks) of alcohol consumed on day used		12.860	.012	8
Psychological health status	16.139		<.001	6
Physical health status	2.828		.052	6
Quality of life	5.689		.003	6
Methamphetamine use disorder participants				
Number of days used amphetamine‐type substance in the last 7 days	4.214		.040	3
Psychological health status	51.000		.001	2
Physical health status	1.370		.384	2
Quality of life	1.190		.435	2

Abbreviation: ATOP, Australian Treatment Outcomes Profile.

###### Clinical characteristics

For the AUD group, breath alcohol content, liver function, blood glucose levels, and urinary drug screen results did not change over the treatment period (Table S3).

##### Methamphetamine use

###### Self‐report

For the MUD group, from baseline to Day 14, the number of days using an amphetamine‐type substance in the past week did not change (Table S4). From baseline to Day 28, the number of days using an amphetamine‐type substance in the past week decreased (Table 3).

###### Clinical characteristics

For the MUD group, liver function, blood glucose levels, urinary drug screen results, and oral fluid test results did not change over time (Table S5).

#### 3.3.2 Craving

**FIGURE 1:**
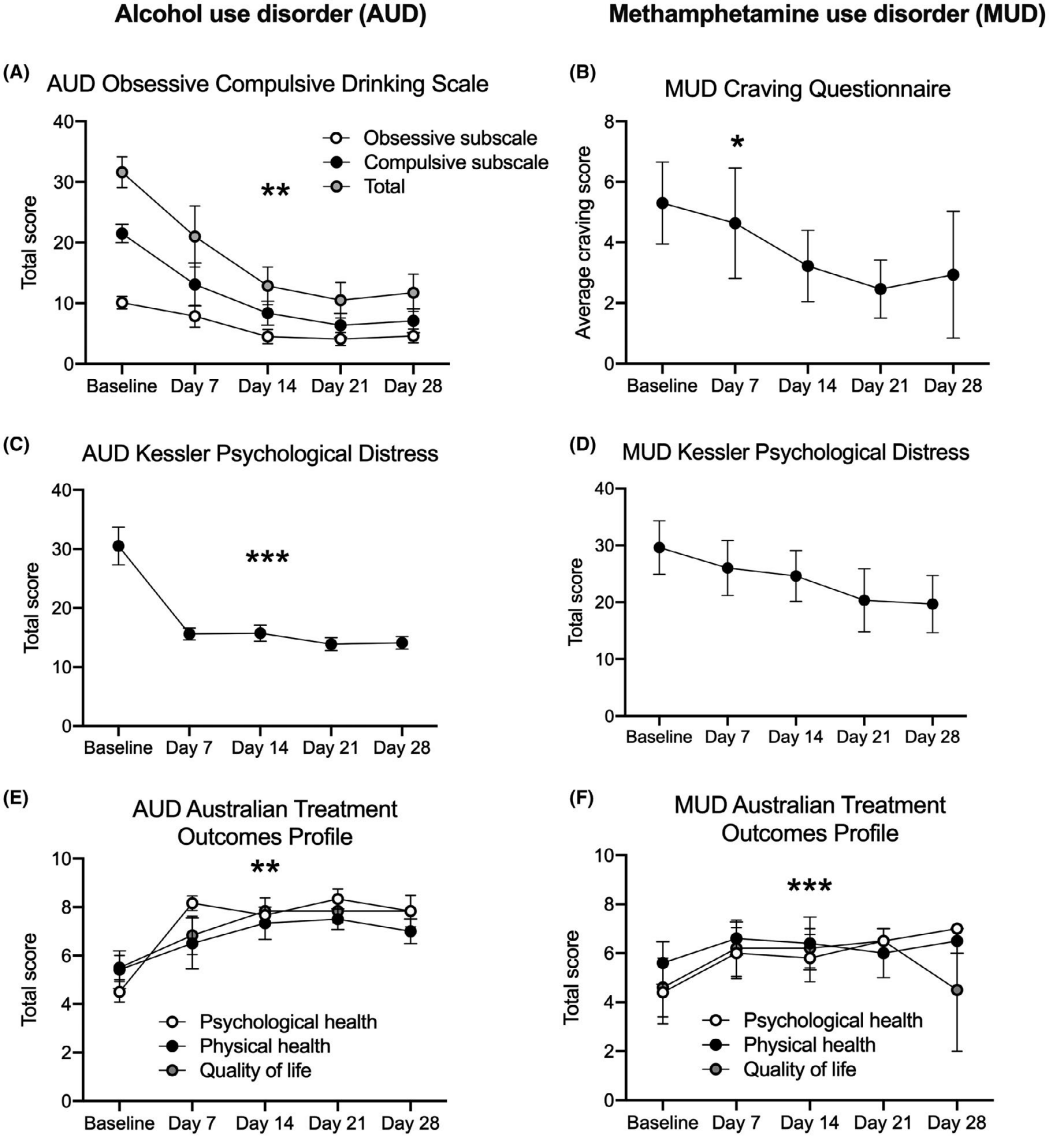
Self‐reported craving, psychological distress, and health and well‐being after lorcaserin treatment in participants with alcohol use disorder (AUD) and methamphetamine use disorder (MUD). Total obsessive compulsive drinking score over the 28‐day treatment period. Total *n* = 8. **p < .01 for each craving measure (A). Average craving score over the 28‐day treatment period. *n* = 3–5. *p < .05 baseline to Day 14 (B). Kessler Psychological Distress self‐report data over the 28‐day treatment period. Total *n* = 8 for AUD participants, *n* = 3–5 for MUD participants. ***p < .001 (C and D). Psychological health status and quality of life increased over the treatment period; however, there was no significant change in physical health status for AUD participants. Total *n* = 8. **p < .01 for psychological health status and quality of life (E). Psychological health status increased over the treatment period; however, there was no significant change in physical health status or quality of life. *n* = 2–5. ***p = 0.001 for psychological health status (F). All data are presented as mean ± SEM

Total craving scores for participants with AUD decreased over time (*F*_4, 28_ = 7.299, *p* < .001; Figure 1A). Scores on the obsessive subscale (*F*_4, 28_ = 5.200, *p* = .003) and on the compulsive subscale (*F*_4, 28_ = 7.689, *p* < .001) also significantly decreased over time (Figure 1A).

Methamphetamine craving for participants with MUD significantly decreased from baseline to Day 14 (*F*_2, 8_ = 5.200, *p* = .036) and approached significance from baseline to Day 28 (*F*_4, 8_ = 2.880, *p* = .095; Figure 1B).

#### 3.3.3 K10 psychological distress

K10 psychological distress scores significantly decreased over time for participants with AUD (*F*_4, 28_ = 20.629, *p* < .001; Figure 1C).

K10 psychological distress scores did not change between baseline and Day 14 (*F*_2, 8_ = 4.193, *p* = .057) or between baseline and Day 28 for participants with MUD (*F*_4, 8_ = 1.537, *p* = .280; Figure 1D).

#### 3.3.4 ATOP self‐report health and well‐being data

For the AUD participants, psychological health status and quality of life significantly increased across the experimental period between baseline and Day 28, and physical health status approached a significant improvement (Table 3; Figure 1E).

For the MUD participants, from baseline to Day 14, the perceived health and well‐being in the past week did not change (Table S4). From baseline to Day 28, the psychological health status in the past week increased; however, physical health status and quality of life did not change (Table 3; Figure 1F).

### 3.4 Pharmacokinetics of lorcaserin

**FIGURE 2:**
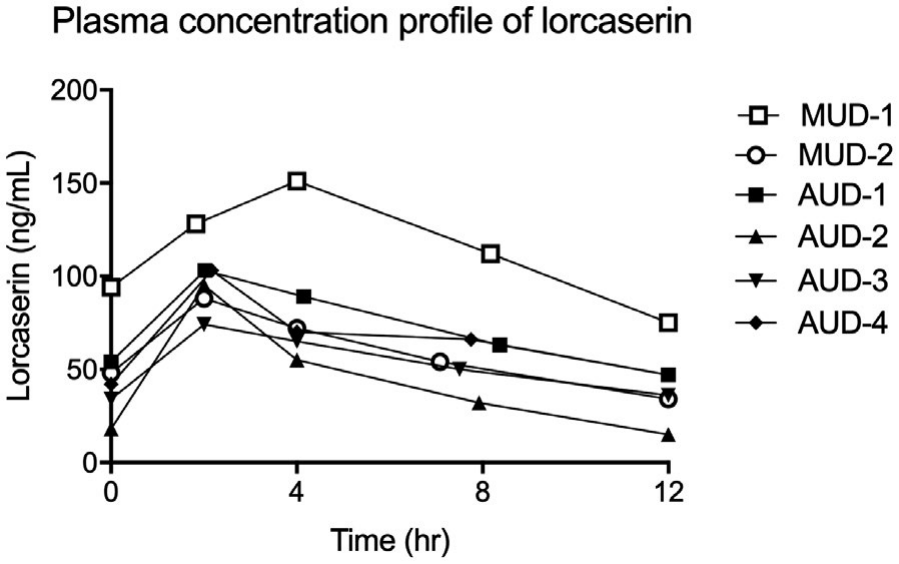
Plasma concentration time profiles (0, 2, 4, 8, and 12 h) after lorcaserin was administered. The pharmacokinetic profile of lorcaserin followed a standard oral absorption profile for the six participants examined. The 12‐h time point for each individual was predicted with a one compartment model using PKSolver.^37^ AUD, alcohol use disorder; MUD, methamphetamine use disorder

Plasma concentration time profiles (0, 2, 4, 8, and 12 h) for six participants are shown in Figure 2. The pharmacokinetic profile followed an oral absorption profile (Figure S1) with a *T*_max_ at 2 h and a *C*_max_ of 102 ± 25 ng/ml before following a one phase exponential decay, resulting in a *t*_1/2_ of 7.9 ± 2 h. Pharmacokinetic parameters are summarized in Table 4.

**TABLE 4:** Pharmacokinetic parameter summary data

Parameter	Unit	Mean	SD	CV (%)
*t*_1/2_	h	7.9	2.0	26
*T*_max_	h	2.4	0.8	34
*C*_max_	ng/ml	102	26	26
AUC 0–12	ng/ml·h	824	311	38
Css	ng/ml	69	26	38

## 4 DISCUSSION

The aim of our pilot study was to examine the safety and efficacy of the 5‐HT_2C_ receptor agonist lorcaserin in people undergoing residential withdrawal and seeking treatment for AUD and MUD. There are limited data on the pharmacokinetics of lorcaserin, and in general, this has been observed in an obese population with a *T*_max_ of 1.5 h, *C*_max_ of 80 ng/ml with a variability of 26%, and a *t*_½_ of 11.9 ± 1.5 h.^40^ In AUD and MUD participants, we observed a slightly longer *T*_max_ of 2.4 h, a higher *C*_max_ of 102 ng/ml with similar variability of 26%, and a shorter *t*_½_ of 7.9 ± 2 h. With the small sample sizes and similar variability of approximately 30% in both studies, it is unlikely that there is a different pharmacokinetic profile between an obese and AUD/MUD populations.

In the present study, AUD participants were recruited faster and had a greater retention rate compared with MUD participants. There were no significant changes in vital signs over the treatment period except respiratory rate did increase for AUD participants. Importantly, lorcaserin was well tolerated, having few, if any, side effects. Lorcaserin was not anorectic and had no cardiovascular side effects. We also tested the effect of lorcaserin on alcohol and Methamphetamine craving and consumption. Although number of drinking days did not change, self‐reported alcohol consumption per session decreased for AUD participants and amphetamine‐type substance use decreased for those MUD participants who we retained. However, there was no change in clinically monitored alcohol or amphetamine content, respectively. Notably, lorcaserin treatment resulted in decreased craving over time for both AUD and MUD participants. Mean baseline craving scores for AUD participants in this study were high compared with those reported for other similar populations ^41^ and decreased by more than 50% at Day 28. MUD participants’ craving scores trended toward significance but showed considerable interparticipant variability. Self‐reported psychological distress scores decreased for AUD participants, but there was no change in psychological distress for MUD participants. Nevertheless, self‐reported psychological health status significantly improved over the treatment period for both AUD and MUD participants. K10 scores for AUD participants dropped to the Australian national average by Day 7.^42^ Overall, and despite the pilot nature of this study, our data do indicate clinically significant improvements on key outcomes and support the notion of 5‐HT_2C_ receptors as a therapeutic target for drug and alcohol abuse. Clinically, 28 days is a short duration of treatment for both alcohol and Methamphetamine dependence. Longer treatment duration may have seen improved outcomes on a number of physiological variables that were trending positively (e.g., GGT levels).

Lorcaserin has shown promise at reducing symptoms of substance use disorder in other, recent clinical trials. For example, lorcaserin reduced cannabis intake compared with placebo controls.^43^ Lorcaserin, in combination with counseling, increased abstinence rates in individuals with nicotine use disorder compared with those treated with smoking cessation counseling alone.^44^ However, lorcaserin did not reduce cocaine or oxycodone use compared with placebo controls.^45^, ^46^ Lorcaserin also did not improve extended‐release naltrexone induction rates in an outpatient sample of individuals with opioid use disorder compared with placebo.^47^ There are some discrepancies regarding the effectiveness of lorcaserin at improving treatment outcomes for those with substance use disorder, but the reasons for these discrepancies are currently unknown and may be due to the mechanistic impact of lorcaserin and the interaction with the drug of choice based on the neural circuits driving the behavior. Nevertheless, at least for alcohol, nicotine, and cannabis, there is supporting evidence.

Our findings are also somewhat consistent with preclinical literature showing reductions in alcohol consumption in rats following chronic lorcaserin treatment.^30^ Lorcaserin can also decrease self‐administration of methamphetamine and cocaine in rhesus monkeys.^29^, ^48^, ^49^ Similarly, lorcaserin decreased heroin self‐administration and cue‐induced reinstatement in rhesus monkeys.^50^, ^51^ Finally, both acute and chronic administration of lorcaserin plus pimavanserin, a selective 5‐HT_2A_ receptor antagonist, decreased cocaine relapse‐like behavior in rats, suggesting that perhaps the actions of the 5‐HT_2C_ receptor alone are not straightforward.^52^

Although lorcaserin was recently withdrawn from the market following a safety concern, we contend the involvement of 5‐HT_2C_ receptors in substance use disorder are relevant and targeting these receptors is still a viable avenue for treatment, particularly given our current findings and those discussed above. The 5‐HT_2C_ receptor has been implicated in both compulsive‐ and impulsive‐like behaviors in rodents, cardinal features of substance use disorder.^53^ Additionally, 5‐HT_2C_ receptor activity can modulate dopamine function.^54^, ^55^ Of note however is the complexity of the 5‐HT system, particularly regarding the interaction between 5‐HT_2C_ and 5‐HT_2A_ receptors and their relationship to impulsive‐like behavior.^56^

### 4.1 Methodological considerations and future research

A methodological limitation of the current study is that all participants received lorcaserin treatment; that is, there was no placebo control. Additionally, this study was open label, limiting the interpretation of our findings. Although overall sample size was small, particularly for MUD participants, those that completed the study seemingly benefitted from lorcaserin. The differential outcomes between AUD and MUD participants go some way to mitigate against these shortfalls; however, further studies are clearly warranted.

MUD participants were more difficult to retain and had lower retention in treatment. The observed difficulty in recruiting and maintaining MUD participants in the trial could possibly be because methamphetamine users seek treatment reluctantly due to a lack of perceived effective treatments. It could also simply reflect that there are more people with AUD than MUD. There was no difference in the rates of AEs between the two groups, so this is unlikely to be a factor. It is difficult to draw firm conclusions due to the small number of participants in the study. Any future study should include design elements to improve recruitment rates and mitigate against attrition. For example, contingency management approaches have been shown to be effective in the treatment of many substance use disorders and hold promise for MUD.^57^, ^58^

## 5 CONCLUSION

In summary, our data suggest that the 5‐HT_2C_ system is still a viable treatment option for substance use disorders, particularly AUD. However, given the current FDA withdrawal of lorcaserin, other 5‐HT_2C_ agonists with high selectivity may be beneficial. Alternatively, due to the conserved nature of the orthosteric binding site for serotonin at 5‐HT_2_ family receptors, selective positive allosteric modulators, analogous to those for acetylcholine muscarinic receptors,^59^ may provide a fruitful line of enquiry and enable more selective targeting. Another promising therapeutic target may be the use of psychedelics, targeting the serotonin system, for substance use disorder treatment.^60^, ^61^ However, animal studies have recently questioned the efficacy of psychedelics in models of AUD.^62^ Nevertheless, our findings add to the literature supporting the 5‐HT_2C_ receptor as a therapeutic target for AUD and MUD.

## DISCLOSURE

The authors report no conflict of interest.

## AUTHOR CONTRIBUTIONS

AJL and YB conceived and designed research; YB, AP, LC, and AN collected the data; EJC analyzed data; PG and JJ conducted and analyzed pharmacokinetic data; EJC and AJL interpreted data and wrote the first draft of the manuscript; all authors contributed to, edited, and approved the final manuscript.

## Supporting information

Supplementary Material

## Data Availability

The data that support the findings of this study are available from the corresponding author upon reasonable request. Some data may not be made available because of privacy or ethical restrictions.
