# Supplementary material for: The 5‐HT2C receptor as a therapeutic target for alcohol and methamphetamine use disorders: A pilot study in treatment‐seeking individuals
Source: Pharmacol Res Perspect. 2021 Apr 30;9(3):e00767. doi: 10.1002/prp2.767 (PMC8085921; doi:10.1002/prp2.767)
Supplement: Supplementary file 1 — Supplementary Material [file PRP2-9-e00767-s001.docx]

**SUPPLEMENTARY DATA**

**THE 5-HT_2C_ RECEPTOR AS A THERAPEUTIC TARGET FOR ALCOHOL AND METHAMPHETAMINE USE DISORDERS: A PROOF OF CONCEPT STUDY IN TREATMENT-SEEKING INDIVIDUALS**

Erin J. Campbell^1*^, Yvonne Bonomo^2*^, Adam Pastor^2^, Lisa Collins^2^, Amanda Norman^2^, Peter Galettis^3^, Janice Johnstone^3^, & Andrew J. Lawrence^1^

^1^ Florey Institute of Neuroscience and Mental Health, The University of Melbourne, Victoria, 3010, Australia.

^2^ St Vincent’s Hospital Melbourne, Department of Addiction Medicine, The University of Melbourne, Victoria 3010, Australia

^3^ School of Medicine and Public Health, The University of Newcastle, New South Wales, 2308, Australia

**Table S1.**

Vital signs from Baseline to Day 28 for participants with alcohol use disorder.

| **Vital signs** | **F statistic** | **P value** | **n** |
| --- | --- | --- | --- |
| Temperature (degrees Celsius) | 2.036 | 0.116 | 8 |
| Pulse (beats/min) | 0.822 | 0.522 | 8 |
| Respiratory (breaths/min) | 3.390 | 0.022 | 8 |
| Blood Pressure: Systolic | 2.035 | 0.117 | 8 |
| Blood Pressure: Diastolic | 1.465 | 0.239 | 8 |
| Weight | 0.725 | 0.583 | 8 |

**Table S2.**

Vital signs from Baseline to Day 14 for participants with methamphetamine use disorder.

| **Vital signs** | **F statistic** | **P value** | **n** |
| --- | --- | --- | --- |
| Temperature (degrees Celsius) | 1.763 | 0.232 | 5 |
| Pulse (beats/min) | 0.105 | 0.902 | 5 |
| Respiratory (breaths/min) | 0.635 | 0.554 | 5 |
| Blood Pressure: Systolic | 0.763 | 0.498 | 5 |
| Blood Pressure: Diastolic | 0.084 | 0.921 | 5 |
| Weight | 2.476 | 0.146 | 5 |

**Table S3.**

Clinical characteristics from Baseline to Day 28 for participants with alcohol use disorder. ALP, alkaline phosphatase; GGT, gamma-glutamyl transferase; ALT, alanine aminotransferase.

| **Clinical characteristics** | **F statistic** | **Chi-square** | **P value** | **n** |
| --- | --- | --- | --- | --- |
| Breath alcohol content | 0.852 |  | 0.505 | 8 |
| Liver function – protein total (g/L) | 0.828 |  | 0.457 | 8 |
| Liver function – albumin (g/L) | 1.538 |  | 0.249 | 8 |
| Liver function – ALP (U/L) | 1.675 |  | 0.223 | 8 |
| Liver function – GGT (U/L) | 2.941 |  | 0.086 | 8 |
| Liver function – ALT (U/L) | 2.459 |  | 0.122 | 8 |
| Liver function – Bilirubin Total (micromole/L) | 0.500 |  | 0.617 | 8 |
| Blood glucose (mmol/L) | 0.309 |  | 0.740 | 8 |
| Urinary drug screen |  | 6.400 | 0.171 | 8 |

**Table S4.**

ATOP self-report data from Baseline to Day 14 for participants with methamphetamine use disorder.

| **ATOP self-report** | **F statistic** | **P value** | **n** |
| --- | --- | --- | --- |
| Number of days used amphetamine type substance in the last 7 days | 2.219 | 0.171 | 5 |
| Psychological health status | 1.594 | 0.261 | 5 |
| Physical health status | 0.677 | 0.535 | 5 |
| Quality of life | 1.590 | 0.262 | 5 |

**Table S5.**

Clinical characteristics from Baseline to Day 14 for participants with methamphetamine use disorder. ALP, alkaline phosphatase; GGT, gamma-glutamyl transferase; ALT, alanine aminotransferase.

| **Clinical characteristics** | **F statistic** | **Chi-square** | **P value** | **n** |
| --- | --- | --- | --- | --- |
| Oral fluid substance test |  | 2.000 | 0.368 | 5 |
| Liver function – protein total (g/L) | 0.014 |  | 0.913 | 4 |
| Liver function – albumin (g/L) | 0.931 |  | 0.406 | 4 |
| Liver function – ALP (U/L) | 2.149 |  | 0.239 | 4 |
| Liver function – GGT (U/L) | 0.900 |  | 0.413 | 4 |
| Liver function – ALT (U/L) | 0.075 |  | 0.802 | 4 |
| Liver function – Bilirubin Total (micromole/L) | 3.659 |  | 0.152 | 4 |
| Blood glucose (mmol/L) | 5.531 |  | 0.100 | 4 |
| Urinary drug screen |  | 2.000 | 0.368 | 2 |

**Figure S1. Average pharmacokinetic profile of lorcaserin over a 12 hour period.** Overall pharmacokinetic plasma profile across the 6 participants followed a standard oral absorption profile. The 12 hour time point was predicted with a one compartment model using PKSolver (Zhang et al., 2010). Data are presented as the mean ± standard error of the mean.
